# Enhancing Machine Learning Potentials through Transfer Learning across Chemical Elements

**DOI:** 10.1021/acs.jcim.5c00293

**Published:** 2025-07-07

**Authors:** Sebastien Röcken, Julija Zavadlav

**Affiliations:** † Professorship of Multiscale Modeling of Fluid Materials, Department of Engineering Physics and Computation, TUM School of Engineering and Design, 9184 Technical University of Munich, Garching 85748, Germany; ‡ Atomistic Modeling Center, Munich Data Science Institute, Technical University of Munich, Garching 85748, Germany

## Abstract

Machine learning potentials (MLPs) can enable simulations of ab initio accuracy at orders of magnitude lower computational cost. However, their effectiveness hinges on the availability of considerable data sets to ensure robust generalization across chemical space and thermodynamic conditions. The generation of such data sets can be labor-intensive, highlighting the need for innovative methods to train MLPs in data-scarce scenarios. Here, we introduce transfer learning of potential energy surfaces between chemically similar elements. Specifically, we leverage the trained MLP for silicon to initialize and expedite the training of an MLP for germanium. Utilizing classical force field and ab initio data sets, we demonstrate that transfer learning surpasses traditional training from scratch in force prediction, leading to more stable simulations and improved temperature transferability. These advantages become even more pronounced as the training data set size decreases. We also observe positive transfer learning effects for most out-of-target properties. Our findings demonstrate that transfer learning across chemical elements is a promising technique for developing accurate and numerically stable MLPs, particularly in a data-scarce regime.

## Graphical abstract

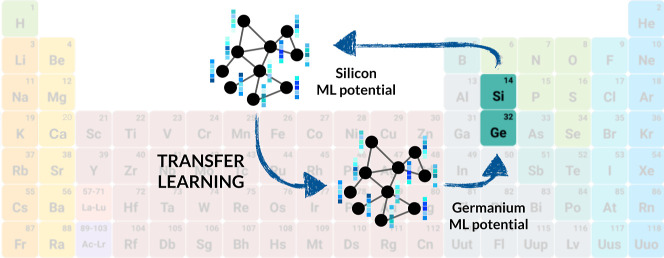

## Introduction

Classical force fields are based on simple, physics-grounded functions, enabling rapid computation of atomic interactions.^1,2^ While they support large-scale molecular dynamics (MD) simulations, they often fall short in terms of accuracy. In contrast, simulations employing approximative solutions to the Schrödinger equation, such as density functional theory (DFT), offer exceptional precision. Unfortunately, their computational demands render them infeasible to explore the extensive spatiotemporal scales typical of many complex systems.

Machine learning potentials (MLPs) have emerged as a powerful tool to reconcile the trade-off between accuracy and computational efficiency.^3–9^ The successful applications in literature span diverse systems ranging from alloys^8,10–13^ to biological macromolecules.^14,15^ Trained on DFT data, MLPs can achieve force prediction errors similar to DFT accuracy^16^ while maintaining the ability to simulate million-atom systems.^5^ Despite these promising results, MLPs are inherently limited by the scope of their training data sets and thus require large and informative data sets to yield reliable and accurate models. Various strategies have been proposed to address challenges related to data scarcity.

One widely used approach is active learning,^17–19^ where new samples are iteratively selected, labeled, and incorporated into the training data set. This process is typically driven by uncertainty quantification criteria, allowing models to identify which data points would be most beneficial for training. However, both data labeling and uncertainty quantification can be computationally intensive, especially when employing rigorous Bayesian methods.^20^ Although non-Bayesian uncertainty quantification techniques^21,22^ may offer viable alternatives with lower computational costs, data labeling remains a notable hurdle in effective active learning implementation.

More recently, there has been a trend toward foundation MLPs,^15,23–29^ where the application domain-unspecific models are built based on extensive data sets comprising a diverse array of chemical elements and compounds. These models can be fine-tuned on a specific chemical subspace, ideally reducing the required training samples. However, this approach mandates a large-scale neural network to capture the interactions between elements of the entire periodic table, resulting in high computational costs for training and inference. As a result, MLPs tailored to narrower chemical spaces or even focused on individual elements present a more computationally efficient alternative. Nevertheless, utilizing the transfer learning concept, the available databases covering different chemical spaces than the downstream application can still provide valuable information.

In transfer learning, the knowledge gained from learning one task can be leveraged and transferred to different but related tasks.^30,31^ If the transferred knowledge provides useful information, it can enhance data efficiency and model accuracy. Therefore, this strategy is particularly advantageous when data for the target task is scarce. Transfer learning has been successfully applied across various domains, from image classification^32^ to materials discovery.^33^ In the context of MLPs, it was used to achieve higher accuracy by pretraining the model on the large DFT data set and fine-tuning it on the more accurate but smaller coupled cluster data set.^34–36^ A similar idea is exploited by Δ-learning, where the difference between a less accurate and a more accurate model is learned.^34^

Contrary to these previous studies, we propose to employ transfer learning across data sets that span different chemical spaces rather than fine-tuning the model on a chemical subspace as in foundational models. Our objective of this work is to leverage the knowledge gained while learning the potential energy surface of a chemical element to enhance the learning of another chemical element. Despite the differences in potential energy surfaces among different chemical elements, the fundamental interaction principles—such as steric and van der Waals forces—are common to all chemical elements. This shared foundation suggests that transfer learning of MLPs across chemical elements could be advantageous, particularly for chemical elements within the same group, as these elements exhibit similar chemical properties. This idea was recently briefly explored by Gardner et al.^37^ However, since they demonstrate the benefits of transfer learning only for force and energy predictions on synthetically altered data, transfer learning analysis is still missing for other properties and for an unaltered real-case data set scenario.

In this paper, we examine transfer learning of MLPs between chemical elements within the carbon group, specifically between silicon and germanium. Our investigation assesses the advantages of transfer learning across two distinct data sets spanning both solid and liquid phases. The first data set utilizes the classical Stillinger-Weber potential, which allows for efficient data generation and enables us to rigorously examine the limits of transfer learning. The second data set is a publicly available DFT data set, presenting a realistic application scenario. Through our analyses of the Stillinger–Weber data set, we demonstrate that transfer learning significantly improves the accuracy of force predictions across the solid and liquid bulk regime, as well as enhances the accuracy of phonon density of states (PDOS) in the small data set regime. Notably, when training models using samples from a single temperature, we observe a marked enhancement in temperature transferability due to transfer learning. In the case of the DFT data set, transfer learning leads to better force prediction accuracy, more stable simulations, and, in some cases, also improved structural properties compared to the models trained from scratch. Overall, our findings highlight the effectiveness of transfer learning across similar chemical elements as a valuable approach for developing MLPs, especially as a tool to overcome the problems of sparse data sets.

## Methods

In this section, we outline the methodology for training MLPs via force matching and describe the procedure for transfer learning among the different chemical elements.

### Machine Learning Potential Training via Force Matching

The MLP architecture utilized in this study is a message-passing graph neural network (GNN) DimeNet++.^38^ This architecture incorporates directional information, which facilitates the inclusion of angular information, thereby enabling precise energy and force predictions. We employ our custom implementation as described in chemtrain.^39^ The embedding size is set to one-fourth of the original, and the cutoff distances are 0.5 nm for Stillinger-Weber data and 0.43 nm for DFT data. The remaining structure adhered to the original implementation.

Different loss functions *L* can be used to optimize the parameters θ of an MLP.^40,41^ Here, we use the force matching loss

1 $$L\left(\theta \right)=\sum_{j=1}^{N_{bs}}\sum_{k=1}^{N}\sum_{l=1}^{3}\frac{1}{3NN_{bs}}{\left(F_{kl}^{T}\left(S_{j}\right)-F_{kl}\left(S_{j};\theta \right)\right)}^{2}$$

where *N*_bs_ is the batch size and *N* is the number of atoms in a configurational state *S*_j_ (3D atomic coordinates and chemical species) in the batch. *F*_*kl*_^T^ and *F*_*kl*_ denote the target and predicted force on atom *k* in the direction *l*, respectively. While it is more common to train also on energy labels, we do not use them here to avoid an additional hyperparameter and more complicated analysis. When training only on forces, the energy is determined only up to a constant. We evaluate this constant after the training by calculating the mean energy shift using the training data. The same constant is then used for energy predictions on the test data set. As detailed in the Supporting Information, we employ the Adam optimizer with a learning rate decay for optimization.

### Transfer Learning

The transfer learning of MLPs across different chemical elements follows a two-stage procedure (Figure 1). In the first stage, we pretrain the MLP on a larger data set containing different structures and corresponding forces of a particular chemical element (e.g., silicon). In the second stage, we use the obtained parameters to initialize the fine-tuning of an MLP for a similar chemical element (e.g., germanium), for which normally only a limited amount of data is available. We compare the performance of the transfer learning models with those trained from scratch that only undergo the second-stage training using random initialization.

**Figure 1:**
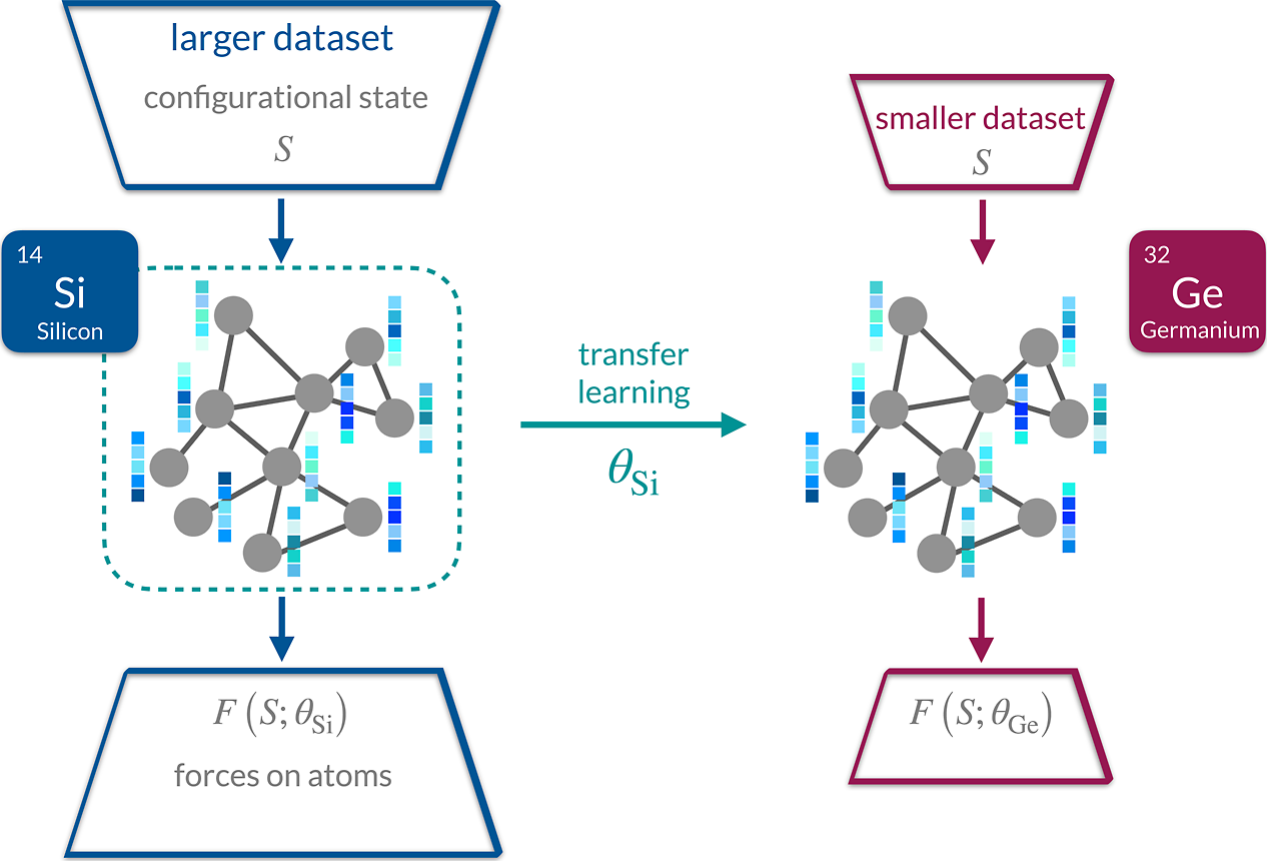
Transfer learning between chemical elements. An MLP is initially trained on a larger data set containing various configurational states ***S*** (3D atomic coordinates and chemical species) and corresponding forces on atoms ***F*** for a certain chemical element, here silicon. The resulting parameters θ are then transferred to initialize the parameters of an MLP, which is subsequently trained on a smaller data set of a similar but different chemical element, here germanium.

In the transfer learning process, it is possible to freeze some parameters. However, our preliminary analysis showed that the best accuracy is achieved when all of the parameters are fine-tuned. Therefore, during the second phase, we retrain all of the parameters of the MLP for the new chemical element. The employed GNN architecture encodes chemical elements via a learnable atom embedding vector. These embedding parameters are also transferred and retrained during the process.

### Data Sets

#### Stillinger-Weber Data Sets

We generate data for silicon and germanium with MD simulations at 34 temperatures in the range 300–3600 K in 100 K intervals. The molecular interactions are parametrized using a Stillinger-Weber potential, following Jian et al.^42^ Simulations of a bulk system comprising 64 atoms are performed in LAMMPS,^43^ employing a time step of 1 fs, a Nose–Hoover thermostat with a damping factor of 100 time steps, and a Nose–Hoover barostat using a damping factor of 1000 time steps. For each temperature, we conducted a 4 ns *NPT* equilibration followed by a 1 ns *NVT* production simulation, sampling every 1 ps. We randomly sample these configurations to create different data sets. For silicon, the train, validation, and test data set contain, respectively, 20, 5, and 10 samples per temperature or 680, 170, and 340 samples in total. For germanium, we discarded the first 100 ps and created a data pool of 30.600 samples (900 per temperature) used for training, validation, and testing. The fact that the training data pool for germanium is larger does not play any role, since the configurations are sampled from equilibrium MD simulations. Critical to the model’s performance is the number of training data points.

#### DFT Data Sets

We utilize the DFT data set for silicon and germanium provided by Zuo et al.^44^ Data include 2 × 2 × 2 bulk structures with deformations up to ±20%, single vacancies, and simulation data at temperatures ranging from 300 K to twice the melting temperature (2422 K for germanium and 3374 K for silicon). We exclude the slab structures from the data set since these are not relevant to our analysis. To avoid exact 180° angles due to code-specific reasons, we add normally distributed noise $N\left(0,10^{-6}\right)$ nm to the particle positions. We partitioned the silicon data into 182 training samples, 20 validation samples, and 23 test samples. For germanium, 24 samples are set apart for testing, while the remaining 217 samples are used as a data pool for training and validation.

For aluminum, we use the data set by Smith et al.,^45^ where we only consider samples containing less than 100 atoms. The data set in the original paper is obtained from an active learning procedure.

### Phonon Density of States

To compute the PDOS, we generate a 2 × 2 × 2 germanium minimum energy supercell with 64 atoms in Avogadro.^46^ For code-specific reasons, we add normally distributed noise $N\left(0,10^{-6}\right)$ nm to the particle positions to avoid exact 180° angles. PDOS is then calculated with phonopy^47,48^ using displacements of 0.01 Å.

### Structural Properties

To evaluate structural properties of the models trained on DFT data, we run 100 MD simulations (20 velocity initializations for each of the five models differing in the train/validation data split). The simulations are performed under *NVT* conditions for 100 ps using the Velocity Verlet integration^49^ and a time step of 0.5 fs. The 1200 K temperature is maintained with the Langevin thermostat^50^ with a damping coefficient of 1/ps. All simulations start with the same liquid state configuration in the data set containing 64 atoms.

## Results

We evaluate the efficacy of transfer learning MLPs across different chemical elements using Stillinger-Weber and DFT data sets containing bulk silicon or germanium structures. The Stillinger-Weber data serves as a test example due to the simple potential energy surface and the ease of data generation, enabling accurate error estimation due to large test data sets. On the other hand, the DFT data provides a realistic application scenario. In all cases, the direction of knowledge transfer is from silicon to germanium.

### Surrogate Model for Stillinger-Weber Potential

#### Data Efficiency

To assess the benefits of transfer learning, we compare the accuracy of the transfer learning models with models trained from scratch for various training data set sizes. The transfer learning models are first pretrained on the silicon data set. On the test data set, the silicon MLP model achieves very low energy and force mean absolute error (MAE) of 0.127 and 0.69 meV/Å, signaling an extensive training data size and a simple target molecular interaction. We then train (retrain in the case of transfer learning) several models on different numbers of samples from the germanium data pool to obtain the germanium MLPs.

In Figure 2, we report the average MAE of energy and force predictions evaluated on a fixed test data set containing 340 configurations within the temperature range of 300–3600 K (10 samples per temperature). Transfer learning consistently outperforms training from scratch across all training sample sizes. The same holds true when comparing transfer learning to the delta learning baseline model. In delta learning, we use the pretrained silicon model as a fixed prior model and train an MLP to match the difference between the prior silicon and the target germanium forces. We observe a significant positive transfer learning effect when germanium data is sparse. For example, transfer learning models trained with just 10 samples achieve a lower MAE than models trained from scratch using 50 samples. We attribute this improvement to the similarity in the two underlying potentials (see Supporting Information for the parameters) and the broad distribution of silicon data. We repeated the analysis also for the solid state samples (Supporting Information) and found the same conclusions.

**Figure 2:**
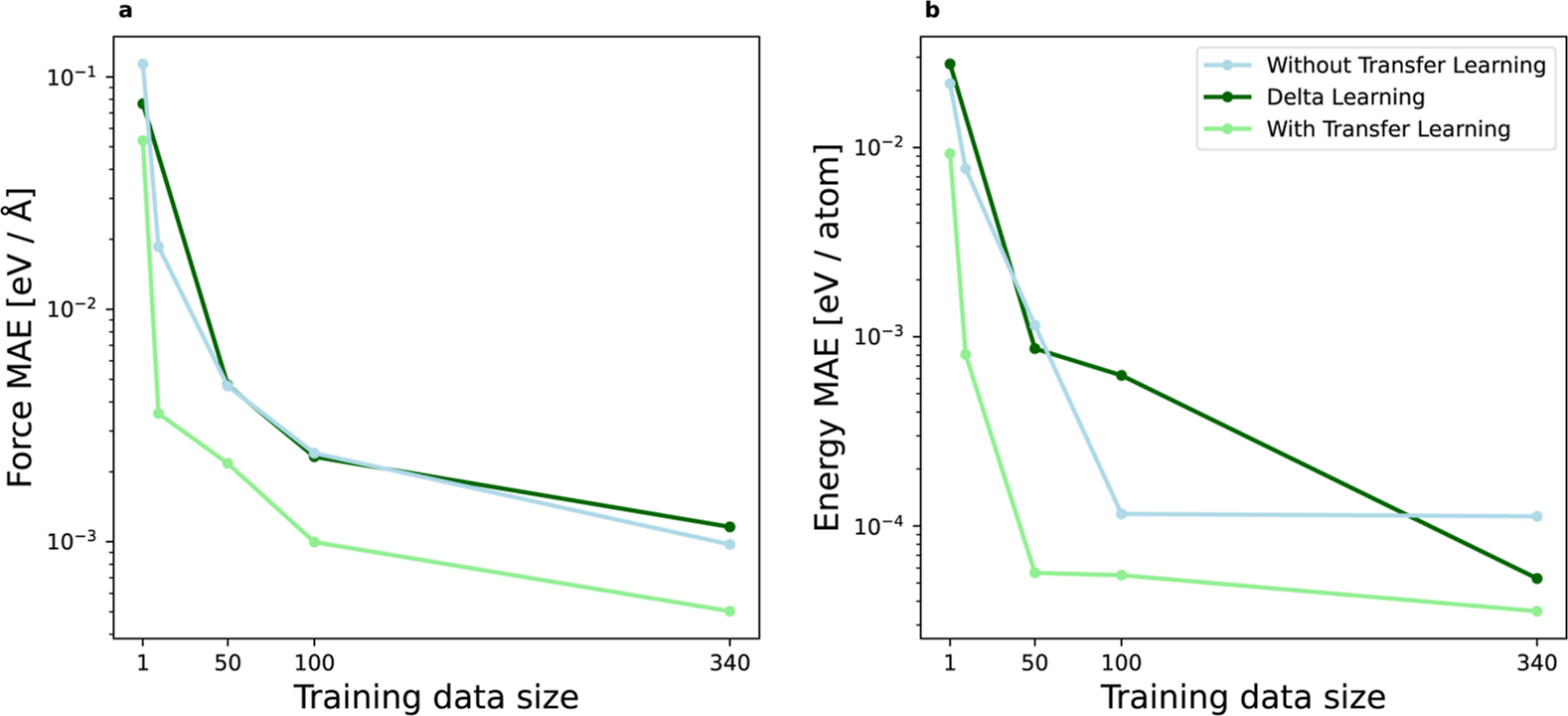
Data efficiency of transfer learning for Stillinger-Weber example. The green and blue lines denote the test set MAE of force (a) and energy (b) predictions for the MLPs trained with and without transfer learning, respectively. An additional baseline model obtained with a delta learning approach is shown with the dark green line. The MAE values are averaged over five different models corresponding to different randomly selected train and validation data samples from the data pool. We perform a hyperparameter search for each model based on the validation data set (170 samples, 5 per temperature) as reported in the Supporting Information.

Next, we consider the performance for out-of-target properties and distributions. To achieve this, we further analyze the models trained with just one sample, as these models are already below the chemical accuracy threshold, both with and without transfer learning. Conversely, we will use the best model trained from scratch with the largest data set of 340 samples as our reference model. At this training data size, the MAEs have converged (Figure 2), indicating that increasing the training data set further would not significantly decrease the MAE.

#### Material Properties

As an out-of-target property, we evaluate the PDOS for MLP models trained on a single random snapshot at 2000 K. These configurations have an amorphous structure, even though the temperature is above the germanium melting point, due to the inaccuracies of the Stillinger-Weber potential and limited sampling. To compute the PDOS, we use phonopy,^47,48^ as outlined in the Methods section.

The results illustrated in Figure 3 demonstrate that models utilizing transfer learning closely match the reference MLP that was trained from scratch on a germanium data set. In contrast, models that do not use transfer learning show significant differences in PDOS compared to the reference, with notable variations among the five models. This outcome is somewhat expected because the minimum energy configuration differs from the amorphous structure used during (re)­training. The enhanced accuracy observed with transfer learning suggests a successful knowledge transfer for the configurational distribution data gaps.

**Figure 3:**
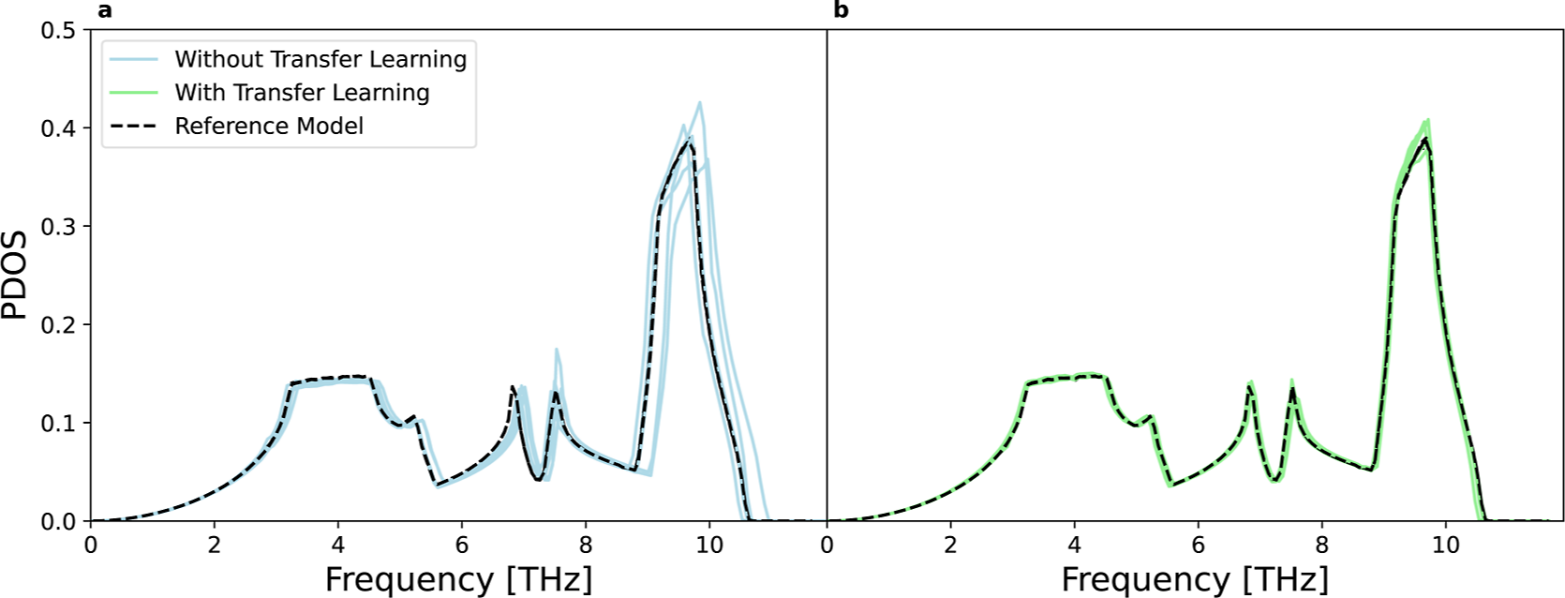
Phonon density of states (PDOS) for Stillinger-Weber example. The results are shown for five models obtained with (b, green) and without (a, blue) transfer learning. These models are trained with a single random sample at 2000 K and compared to the reference germanium model (dashed black) trained on a data set containing 340 samples across the entire considered temperature range. The five models correspond to the five best hyperparameter models with different random selections of training and validation data (5 samples at 2000 K).

#### Temperature Transferability

To further validate this point, we investigate the temperature transferability using the same models as those above trained on a single sample at 2000 K. Figure 4 displays the force MAE per atom across test samples generated in the 300–3300 K temperature range. The transfer learning approach delivers significant gains across the entire temperature range. The silicon MLP model initialization, parametrized with samples ranging from 300 to 3600 K, improves the germanium MLP’s performance in temperature regimes not covered by the single germanium training sample. The same outcome is also observed when training on 10 samples at 2000 K (see Supporting Information).

**Figure 4:**
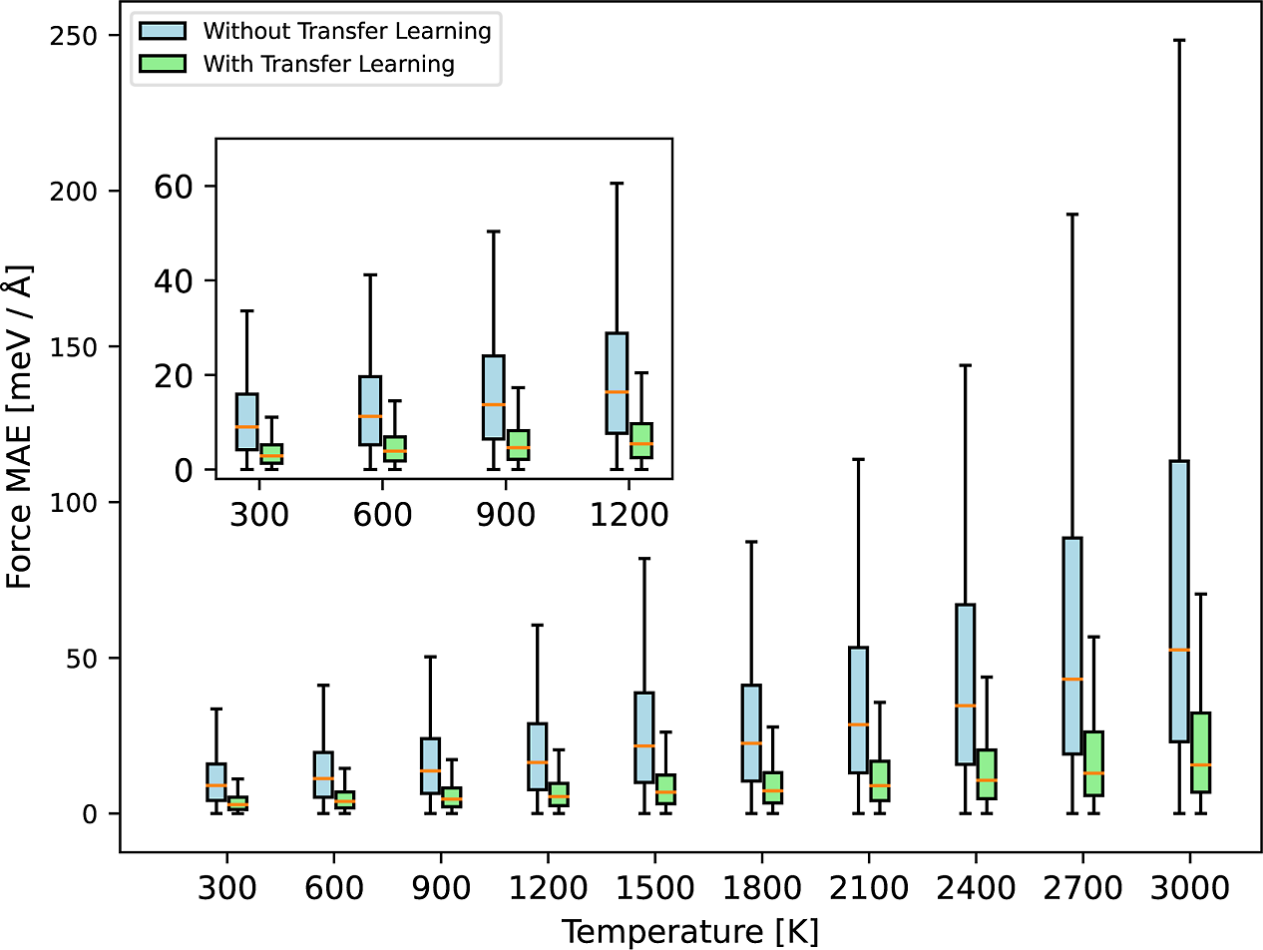
Temperature transferability for Stillinger-Weber example. Transfer learning models (green) are referenced against the models trained from scratch (blue) by computing the force MAE for samples at different temperatures. For both cases, we train five models using a single sample at 2000 K and test each one on 50 samples at the respective temperature, resulting in 250 predictions in total for the five models. On the whisker plot, the orange line indicates the median, the box represents the interquartile range (IQR) between the first and third quartiles, and the whiskers extend to the furthest point within 1.5 times the IQR.

### DFT Surrogate Model

The results presented thus far were based on data generated by using the Stillinger-Weber potential. While this example allowed us to rigorously evaluate performance with extensive test data sets, the Stillinger-Weber potential is relatively simple compared to the more accurate solutions provided by DFT. Thus, we now proceed with a realistic use case by constructing a DFT surrogate model. This model presents an ideal scenario for transfer learning, especially given the challenges associated with generating DFT data.

#### Data Efficiency

We first tested the accuracy of the silicon MLP model. We obtain a similar test set accuracy as Zuo et al.,^44^ confirming an expressive enough GNN and an adequate training strategy. In particular, the energy and force root-mean-square error (RMSE) are 21.8 meV/atom and 0.10 eV/Å, respectively. Zuo et al.^44^ report several models achieving an energy RMSE in the range of 3.02–9.95 meV/atom and a force RMSE in the range of 0.09–0.34 eV/Å. Analogous to the Stillinger-Weber example, we proceed with the data efficiency testing of the transfer learning approach by reporting for germanium MLP models the force and energy MAE using 1, 10, 40, 100, and 195 training samples (Figure 5). Again, we find a significant performance enhancement when employing the transfer learning approach. For the respective training sample sizes, the energy MAE is reduced by 59, 69, 46, 27, and −5% and the force MAE by 38, 29, 16, 6, and 4%, highlighting positive transfer learning effects for scarce data. With only 10 training samples, the transfer learning models achieve an accuracy below the chemical threshold of 43 meV/atom. In contrast, training from scratch requires all of the available data (195 samples) to reach chemical accuracy. Note that at this training data size, the MAE of the models with and without transfer learning is very similar and comparable to the errors reported by Zuo et al.^44^ (RMSE between 3.68–10.96 meV/atom and 0.07–0.29 eV/Å for energy and force, respectively).

**Figure 5:**
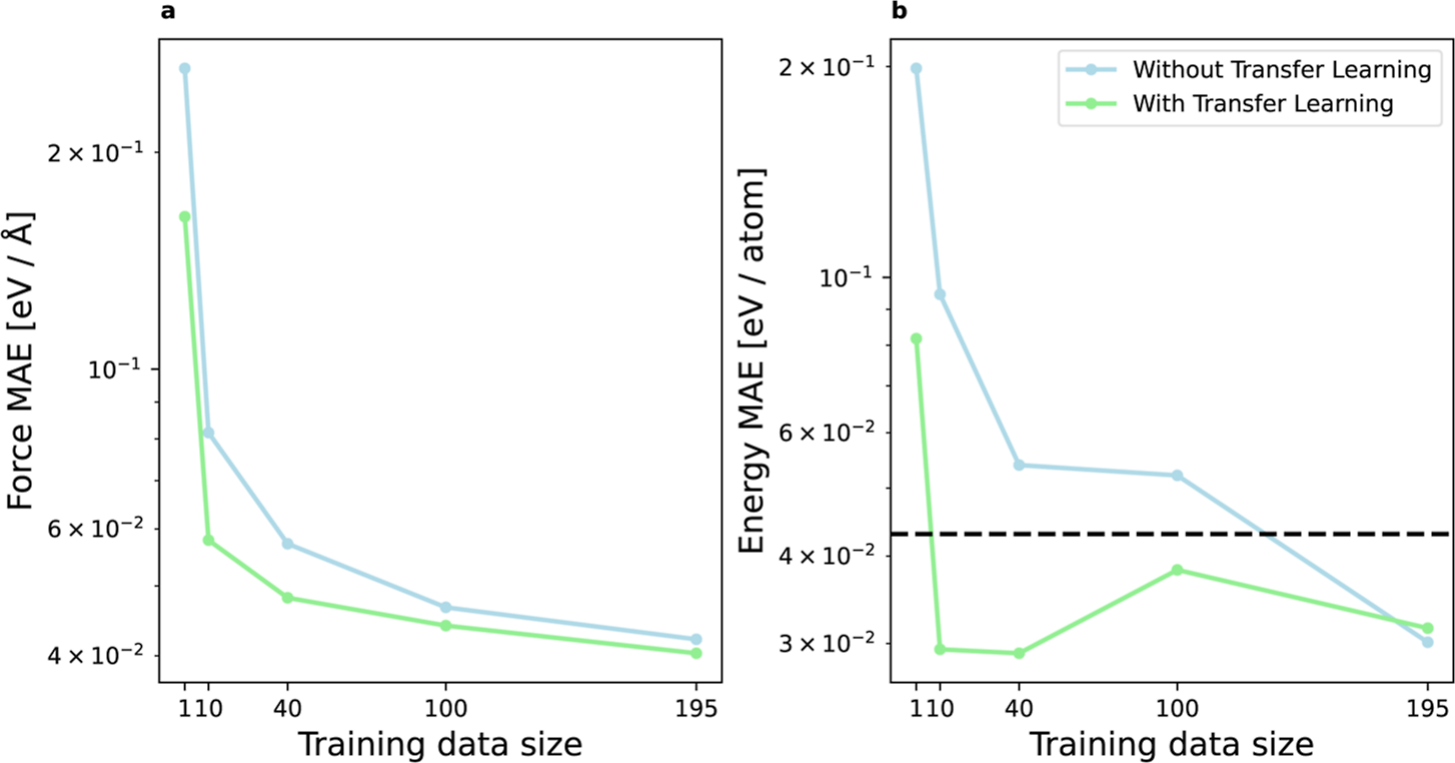
Data efficiency of silicon to germanium transfer learning for the DFT example. The green and blue lines denote the test set MAE of force (a) and energy (b) predictions for the MLPs trained with and without transfer learning, respectively. The MAE values are averaged over five different models corresponding to different randomly selected train and validation data samples. We perform a hyperparameter search for each model based on the validation data set (22 samples) as reported in the Supporting Information. The black dashed line denotes the chemical accuracy of 43 meV/atom.

In addition to evaluating transfer learning between neighboring elements in the same column of the periodic table, we also test the approach for neighboring elements in the same row of the periodic table, i.e., by replacing germanium with aluminum. Also in this case, transfer learning outperforms learning from scratch for all tested training data set sizes (see Figure S4). However, the positive effect is less pronounced, which could be due to the different crystal structures of silicon and aluminum or due to the discrepancies in the DFT data set construction and labeling.

#### Structural Properties

We further assess the impact of transfer learning by examining the structural properties of liquid germanium. In particular, the radial distribution functions (RDFs) and angular distribution functions (ADFs) are shown in Figure 6. The structural properties are evaluated from 100 forward simulations of 100 ps length, as detailed in the Methods section.

**Figure 6:**
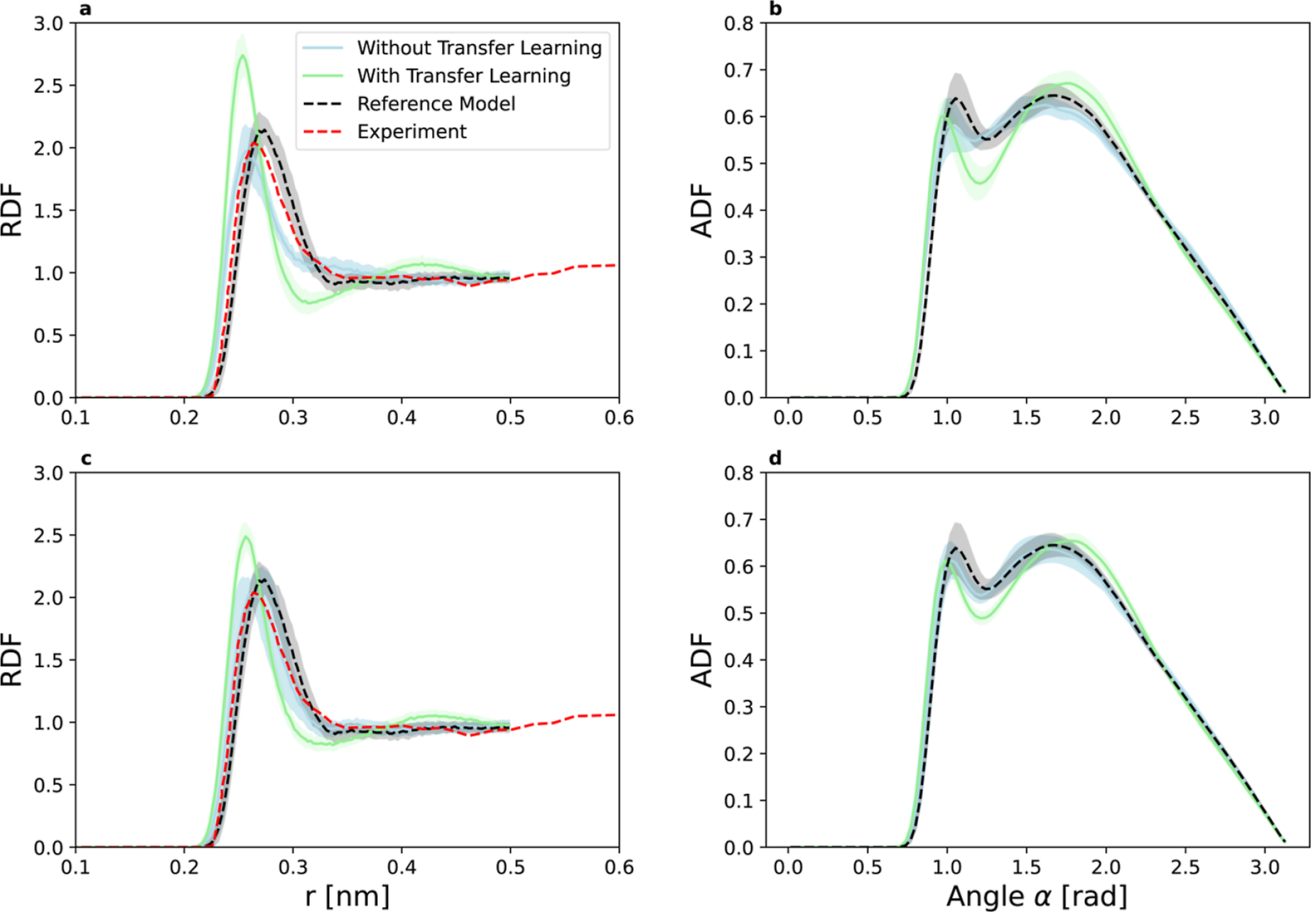
Radial distribution function (RDF; left) and angular distribution function (ADF; right) for the silicon to germanium DFT example and training data size of 10 (a,b) and 100 (c,d). The green and blue lines denote the models employing transfer learning and the models trained from scratch, respectively. As a reference, we show the results also for the reference model (dashed black), i.e., a germanium MLP trained from scratch with all available data (195 samples). Since some simulation runs resulted in unphysical trajectories, we computed the RDF and ADF from states sampled in the time regime of 10–100 ps, only for models that ran for the full 100 ps and yielded zero RDF values below 0.2 nm. The dashed red line represents experimental results from neutron diffraction studies at 1273.15 K.^51^ Shaded regions denote the standard deviation.

With scarce data, the structural properties are not yet fully converged for both approaches—those that use transfer learning and those that do not. Additionally, the RDFs show some deviation from the experimental curve, even with the largest training data set. This suggests either inaccuracies in the DFT calculations or insufficient data. Interestingly, models trained from scratch display a closer alignment with the experimental data, revealing a negative transfer learning effect for the structural properties, despite having lower errors in energy and force predictions. For models that utilize transfer learning, the RDFs and ADFs gradually shift from the values obtained using the pretrained silicon model to those derived from the reference germanium model trained from scratch (see Supporting Information, Figure S3, which uses simulations at 2000 K). This finding effectively illustrates the mechanics of transfer learning: the models revert to the pretrained solution in regions where training data is limited. Although the pretrained silicon model differs from the target germanium model, it is physically valid and contributes to improved numerical stability, as demonstrated in the following section.

Contrary to the results presented for germanium, we observe positive transfer learning for aluminum with respect to the structural properties. The transfer-learned models match the position of the first RDF peak better than the corresponding models trained from scratch (Figure 7). We attribute this difference to the differences in the construction of the data sets. The aluminum data set was generated with an active learning procedure by performing MD simulations at different temperatures starting from random disordered configurations. On the other hand, a large portion of the germanium data set contains deformed crystal structures, which are not as relevant to the liquid state’s structural properties.

**Figure 7:**
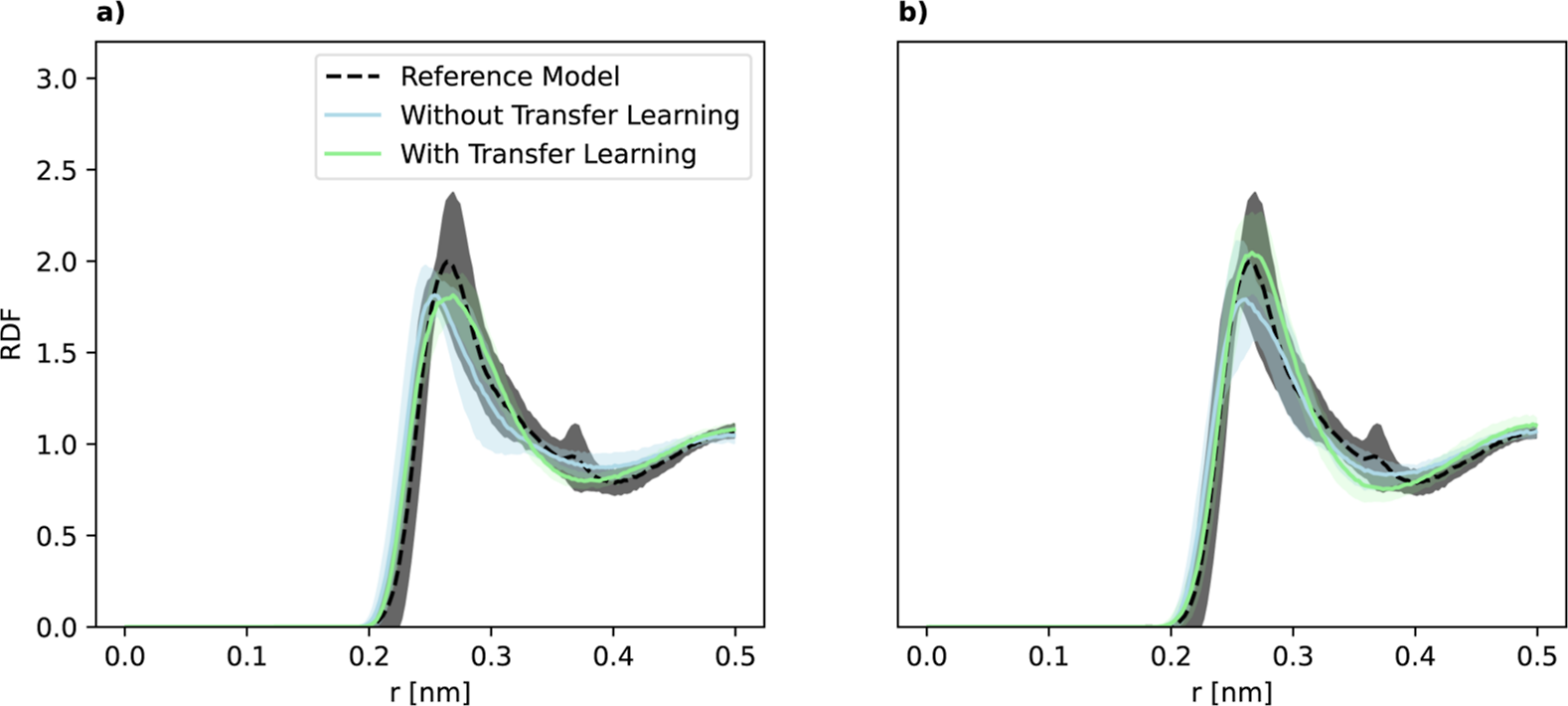
Radial distribution function (RDF) for the silicon to aluminum DFT example at 2000 K and training data size of 10 (a) and 50 (b). The green and blue lines denote the models employing transfer learning and the models trained from scratch, respectively. As a reference, we also show the results for the reference model (dashed black), i.e., an aluminum MLP trained from scratch with 200 samples. We computed the RDF from states sampled in the time regime of 10–100 ps. Shaded regions denote the standard deviation.

#### Numerical Stability

Over the past decade, the development of MLPs has advanced significantly. However, numerical stability remains a crucial area that requires improvement.^52^ Specifically, MLPs can display pathological behavior, such as extreme energy and force predictions, resulting in unphysical trajectories characterized by unrealistic bond breaking, particle overlap, and unstable simulations that may lead to simulation failure.

The stability of MLPs is typically assessed using structural criteria, such as measuring deviations from equilibrium RDFs or equilibrium bond lengths.^52^ In our analysis, we examined 100 forward trajectories used for RDF and ADF computations and evaluated the number of simulations that successfully completed 100 ps without any RDF values falling below 0.2 nm, which signifies no particle overlaps (Table 1).

**Table 1:** Numerical Stability of DFT Surrogate Models with and without Transfer Learning from Silicon to Germanium and Various Training Data Sizes^a^

approach	training data size			
	10	40	100	195
without transfer learning	35	46	73	71
with transfer learning	100	100	80	80

a The reported values correspond to the number of successful simulations out of 100 that reached 100 ps without exploding and displaying a zero RDF value below 0.2 nm.

We find that transfer learning significantly improves the numerical stability of MLPs, particularly when working with small training data sets. When forward simulations of liquid germanium are conducted using MLPs trained on limited samples, we are likely to encounter out-of-distribution states. However, by employing transfer learning from a silicon MLP with a broader training data distribution, we can access approximate and physically sound information in these unseen regimes, resulting in more stable behavior. Similar results are observed for the aluminum case. For one training sample, models trained without transfer learning are successful in 51/100 simulations, while models employing transfer learning are stable in all simulations. For 10, 50, and 100 training samples, both with and without transfer learning, yield 100% stable simulations.

Our findings support the overarching hypothesis that pretraining the model on a chemically similar system allows for the transfer and preservation of information from the pretraining data set, which is absent from the fine-tuning data set. The germanium transfer learning models are slightly less stable with increased training data set size. This behavior could be due to differences in the silicon and germanium data sets, given that it is not observed for aluminum models. The positive impacts of transfer learning are thus most pronounced when there is a significant difference between the distributions of the pretraining and fine-tuning data sets.

## Conclusions

In conclusion, our study highlighted the advantages of the transfer learning technique in the development of MLPs. We built upon the idea that the underlying physical principles governing the interactions of atoms are shared. The more similar two chemical elements are, the closer their potential energy surfaces are likely to be. This reasoning underpins the assumption that transfer learning is beneficial for training MLPs between similar chemical elements. Indeed, we found many benefits of transfer learning MLPs from silicon to germanium, which are closely related and share the same crystal structure. It enables significantly higher accuracy in force and energy predictions in scenarios with limited training data, supporting the hypothesis that transfer learning among similar chemical elements enhances data efficiency and accuracy. This is especially relevant for MLPs targeting a high, e.g., CCSD­(T) (coupled cluster using single, double, and perturbative triple excitations^53^), level of accuracy. Additionally, we present examples of both positive and negative transfer learning outcomes for other property predictions, allowing for a deeper understanding of the transfer learning mechanism. Importantly, we demonstrate that transfer learning facilitates more stable simulations. This indicates that the silicon potential energy landscape information is transferred in the process, providing an approximate, yet physically sound, solution in the configurational space unseen during stage two training. Transfer learning could therefore play a key role in overcoming numerical stability challenges associated with MLPs.

## Supplementary Material



## Data Availability

The data that supports the findings of this study are available within the article and its Supporting Information. The code to train presented models will be open-sourced at GitHub: https://github.com/tummfm/TL_MLP.git.

## Notes

Stillinger-Weber potential parameters; hyperparameter for training on Stillinger-Weber data; hyperparameter for training on DFT data; data efficiency on test samples in the 300–900 K range for Stillinger-Weber data; temperature transferability when training on 10 samples for Stillinger-Weber data; structural properties for simulations at 2000 K; and data efficiency of transfer learning from silicon to aluminum (PDF)
