# Supplementary material for: Enhancing Machine Learning Potentials through Transfer Learning across Chemical Elements
Source: J Chem Inf Model. 2025 Jul 7;65(14):7406–14. doi: 10.1021/acs.jcim.5c00293 (PMC12308786; doi:10.1021/acs.jcim.5c00293)
Supplement: Supplementary file 1 [file ci5c00293_si_001.pdf]

Supplementary - Enhancing Machine Learning Potentials through Transfer Learning across Chemical Elements

Sebastien Röcken[†] and Julija Zavadlav[*,†,‡]

†*Professorship of Multiscale Modeling of Fluid Materials, Department of Engineering Physics and Computation, TUM School of Engineering and Design, Technical University of Munich, Germany*

‡*Atomistic Modeling Center, Munich Data Science Institute, Technical University of Munich, Germany*

E-mail: julija.zavadlav@tum.de

Methods

Energy Evaluation

We train our MLP on forces only. However, in our implementation, the forces are derived from the potential energy. By integrating the derivative of the potential energy, we get back the energy plus an integration constant. This implies that if we train on forces, we learn the energy up to an integration constant. In testing our framework, we evaluate the energies of the test set by first evaluating the constant energy shift our model predicts on the training data, and then subtracting this constant energy shift from the predicted energies of the test set.

Additional Results

Table S1: Parametrization of the Stillinger-Weber potential for silicon? and germanium? as summarized by Jian et al.[1]

Hyperparameter	A	B	ϵ	σ	λ	δ	p	q	γ	b
Silicon model	7.04956	0.60222	2.1702	2.0951	21.0	1.0	4.0	0.0	1.2	1.8
Germanium models	7.04956	0.60222	1.93	2.181	31.0	1.0	4.0	0.0	1.2	1.8

Table S2: Hyperparameter search for MLPs trained on Stillinger-Weber data. The germanium models with and without transfer learning are trained on five different seeds (data shuffle) for each hyperparameter set and the best model is picked based on validation data. The pre-trained silicon model is trained on a single seed.

Hyperparameter	Initial learning rate	Learning rate decay	Epochs
Silicon model	1e-2, 5e-3, 1e-3	1e-4, 5e-4, 1e-3	750
Germanium models	1e-2, 5e-3, 1e-3	1e-4, 5e-4, 1e-3	1000
	5e-4, 1e-4, 5e-5	5e-3, 1e-2, 5e-2	

Table S3: Hyperparameter search for MLPs trained on DFT data. The germanium and aluminum models with and without transfer learning are trained on five different seeds (data shuffle) for each hyperparameter set and the best model is picked based on validation data. The pre-trained silicon model is trained on a single seed.

Hyperparameter	Initial learning rate	Learning rate decay	Epochs
Silicon/germanium/aluminum models	1e-2, 5e-3, 1e-3	1e-4, 5e-4, 1e-3	500
	5e-4, 1e-4, 5e-5	5e-3, 1e-2, 5e-2	500

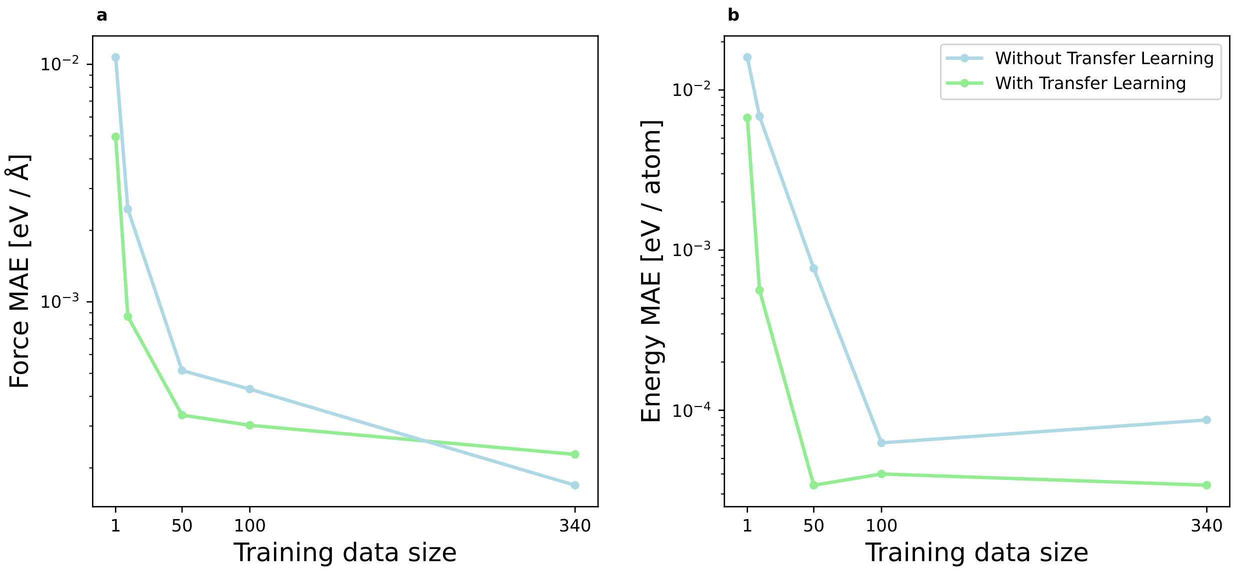

Figure S1: Data efficiency of transfer learning for Stillinger-Weber example using solid state data subset. The test set consists of 70 samples, spanning the temperature range of 300-900 K, with 10 samples per temperature. All other details are the same as in Figure 2 of the main article.

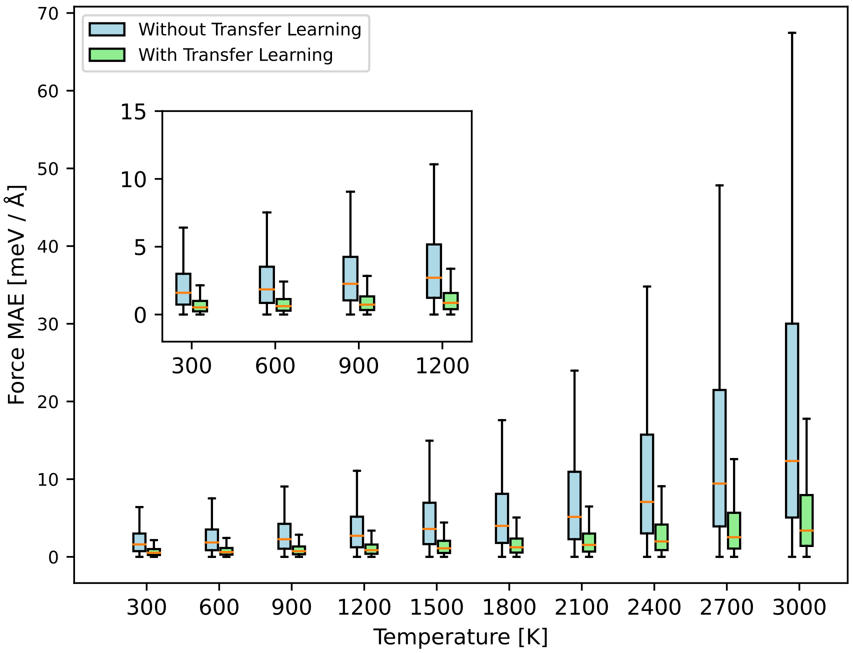

Figure S2: Temperature transferability for Stillinger-Weber example using germanium training dataset of 10 samples at 2000 K. All other details are the same as in Figure 4 of the main article.

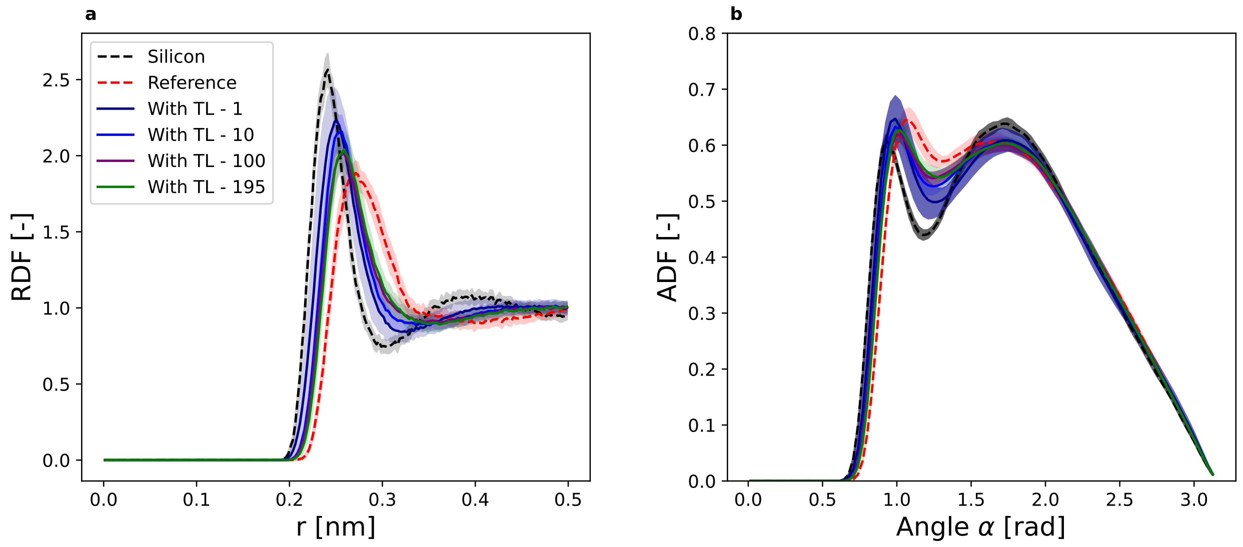

Figure S3: Radial Distribution Function (RDF; left) and Angular Distribution Function (ADF; right) for DFT surrogate models obtained with 2000 K simulations initiated with a liquid germanium configuration. Silicon model denotes the pre-trained MLP, Reference mode denotes the model trained on 195 germanium samples without transfer learning, and With TL - [1, 10, 100, 195] denotes training on [1, 10, 100, 1995] germanium samples using transfer learning. The results are shown for stable 100 ps simulations with zero RDF values below 0.16 nm.

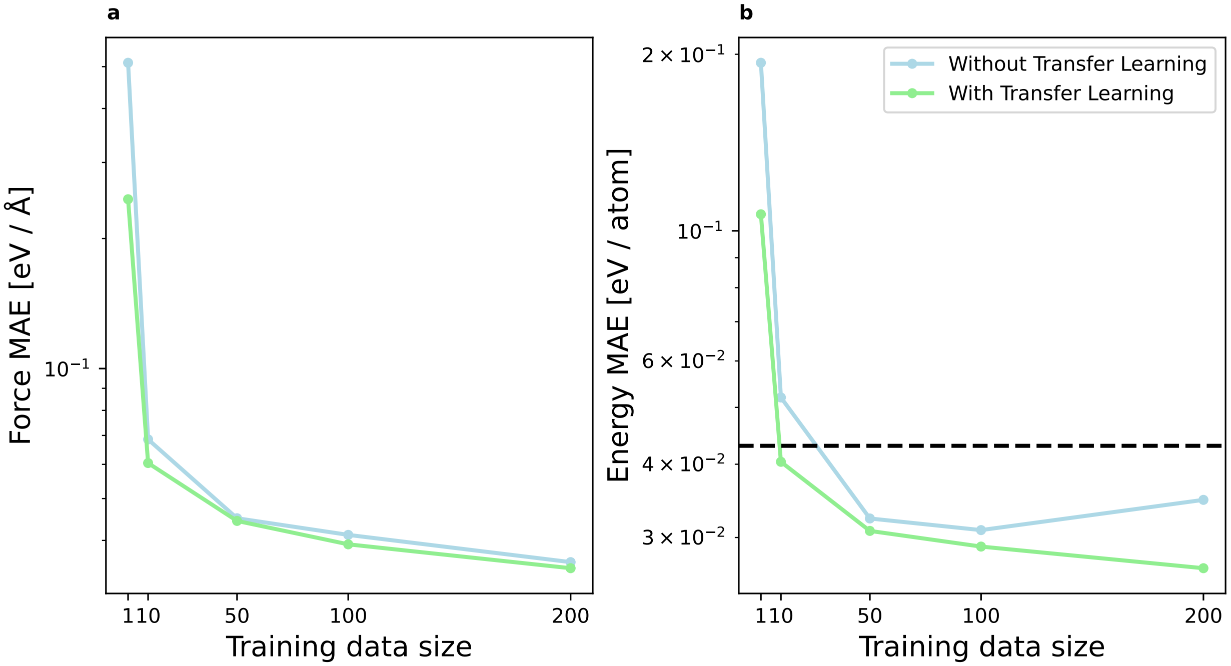

Figure S4: Data efficiency of transfer learning from silicon to aluminium using DFT dataset by Smith et al.,[2] where we only consider samples containing less than 100 atoms. The green and blue lines denote the test set (162 samples) MAE of force (a) and energy (b) predictions for the MLPs trained with and without transfer learning, respectively. The MAE values are averaged over five different models corresponding to different randomly selected train and validation data samples. We perform a hyperparameter search for each model based on the validation dataset (25 samples). The hyperparameter search is done the same way as for silicon and germanium.

References

(1) Jian, Z.; Kaiming, Z.; Xide, X. Modification of stillinger-weber potentials for si and ge. Physical Review B **1990**, 41, 12915.

(2) Smith, J. S.; Nebgen, B.; Mathew, N.; Chen, J.; Lubbers, N.; Burakovsky, L.; Tretiak, S.; Nam, H. A.; Germann, T.; Fensin, S.; others Automated discovery of a robust interatomic potential for aluminum. Nature communications **2021**, 12, 1257.
